# The Gut-Associated Lymphoid Tissues in the Small Intestine, Not the Large Intestine, Play a Major Role in Oral Prion Disease Pathogenesis

**DOI:** 10.1128/JVI.01544-15

**Published:** 2015-08-19

**Authors:** David S. Donaldson, Kathryn J. Else, Neil A. Mabbott

**Affiliations:** a The Roslin Institute & Royal (Dick) School of Veterinary Sciences, University of Edinburgh, Easter Bush, United Kingdom; b Faculty of Life Sciences, University of Manchester, Manchester, United Kingdom

## Abstract

Prion diseases are infectious neurodegenerative disorders characterized by accumulations of abnormally folded cellular prion protein in affected tissues. Many natural prion diseases are acquired orally, and following exposure, the early replication of some prion isolates upon follicular dendritic cells (FDC) within gut-associated lymphoid tissues (GALT) is important for the efficient spread of disease to the brain (neuroinvasion). Prion detection within large intestinal GALT biopsy specimens has been used to estimate human and animal disease prevalence. However, the relative contributions of the small and large intestinal GALT to oral prion pathogenesis were unknown. To address this issue, we created mice that specifically lacked FDC-containing GALT only in the small intestine. Our data show that oral prion disease susceptibility was dramatically reduced in mice lacking small intestinal GALT. Although these mice had FDC-containing GALT throughout their large intestines, these tissues were not early sites of prion accumulation or neuroinvasion. We also determined whether pathology specifically within the large intestine might influence prion pathogenesis. Congruent infection with the nematode parasite Trichuris muris in the large intestine around the time of oral prion exposure did not affect disease pathogenesis. Together, these data demonstrate that the small intestinal GALT are the major early sites of prion accumulation and neuroinvasion after oral exposure. This has important implications for our understanding of the factors that influence the risk of infection and the preclinical diagnosis of disease.

**IMPORTANCE** Many natural prion diseases are acquired orally. After exposure, the accumulation of some prion diseases in the gut-associated lymphoid tissues (GALT) is important for efficient spread of disease to the brain. However, the relative contributions of GALT in the small and large intestines to oral prion pathogenesis were unknown. We show that the small intestinal GALT are the essential early sites of prion accumulation. Furthermore, congruent infection with a large intestinal helminth (worm) around the time of oral prion exposure did not affect disease pathogenesis. This is important for our understanding of the factors that influence the risk of prion infection and the preclinical diagnosis of disease. The detection of prions within large intestinal GALT biopsy specimens has been used to estimate human and animal disease prevalence. However, our data suggest that using these biopsy specimens may miss individuals in the early stages of oral prion infection and significantly underestimate the disease prevalence.

## INTRODUCTION

Prion diseases (transmissible spongiform encephalopathies [TSEs]) are subacute neurodegenerative diseases affecting both animals and humans and are characterized by the accumulation of aggregations of PrP^Sc^, abnormally folded isoforms of the cellular prion protein (PrP^C^), in affected tissues. Infectivity copurifies with PrP^Sc^, and it appears to constitute the major, if not sole, component of the infectious agent (1). Many prion diseases, including natural sheep scrapie, bovine spongiform encephalopathy, chronic wasting disease (CWD) in mule deer and elk, and kuru and variant Creutzfeldt-Jakob disease (vCJD) in humans, are acquired peripherally by oral consumption of prion-contaminated food.

The gut-associated lymphoid tissues (GALT) comprise a collection of multifollicular structures, including the tonsils, Peyer's patches, appendix, colonic and cecal patches, and a number of smaller, single follicular structures termed isolated lymphoid follicles (ILF). These tissues are situated throughout the gastrointestinal tract, and together with the mesenteric lymph nodes (MLN), they help protect the host from infection. However, following oral exposure, some prion isolates exploit the GALT to infect the host (2–4), where they replicate upon follicular dendritic cells (FDC) in the B-cell follicles before spreading via enteric nerves to the central nervous system (CNS) (a process termed neuroinvasion) (2–7). Once the prions have been amplified on the surfaces of FDC above the threshold required for neuroinvasion, they subsequently infect the enteric nerves within the intestine (8, 9). The prions then spread through the peripheral nervous system (both sympathetic and parasympathetic) and infect the CNS (10, 11), although hematogenous spread cannot be entirely excluded. Our previous data suggest that neuroinvasion after oral exposure occurs directly via GALT since neuroinvasion was blocked in mice that lacked GALT (3).

The ILF in the intestine can be classified as either immature ILF (individual primary B-cell follicles) or mature ILF containing a single organized B-cell-containing germinal center and an FDC network (12–16). We have shown that FDC-containing mature ILF were a novel, previously unrecognized site of prion accumulation and neuroinvasion in the intestine. Mice that lacked organized patch-like structures such as the Peyer's patches but contained numerous FDC-containing ILF throughout their intestines displayed unaltered prion disease pathogenesis and susceptibility after oral exposure compared to intact control mice (3).

Prions accumulate in both small intestinal (SI) and large intestinal (LI) GALT. Accumulation within LI GALT, such as the rectoanal mucosa-associated lymphoid tissues (RAMALT) of scrapie- and CWD-affected species (17, 18) and the appendix of vCJD-affected humans, has received significant attention, as it has been used to identify preclinical infected animals and to gain insight into the possible prevalence of vCJD in the United Kingdom (19, 20). However, the relative contribution of LI GALT in oral prion disease susceptibility has been mostly overlooked as prion uptake studies have focused on the uptake of prions directly into Peyer's patches in the SI or have analyzed tissues collected toward the clinical stage of disease, after neuroinvasion has occurred. Importantly, in cases where LI GALT has been studied in natural host species earlier in disease, it appears that the prion accumulation within these tissues may occur secondary to that of SI GALT (21–23). While this may relate in part to sensitivity of detection, it questions the reliability of sampling LI GALT as a prion diagnostic. Therefore, in this study, mice that were specifically deficient in FDC-containing GALT only in the SI were created. These were then used to determine whether the GALT in the SI or the LI were the important sites of early prion accumulation and subsequent neuroinvasion after oral exposure. Since the colon is the major colonization site for commensal bacteria, disturbances to the gut microbiota or inflammation or pathology within the mucosa or the GALT in the LI may have significant influence on oral prion disease pathogenesis. Therefore, we also determined whether the pathology or inflammation caused by a congruent pathogen infection that was specifically restricted to the LI may influence oral prion disease pathogenesis.

## MATERIALS AND METHODS

### Mice.

C57BL/6J mice were used throughout this study and maintained under specific-pathogen-free (SPF) conditions. All studies and regulatory licenses were approved by the University of Edinburgh's ethics committee and carried out under the authority of a UK Home Office Project License.

### *In utero* LTβR-blockade.

Pregnant C57BL/6J mice were injected intravenously (i.v.) with 100 μg of lymphotoxin β receptor (LTβR)-Ig (Biogen Idec, Weston, MA, USA) (24) on embryonic day 11.5 (E11.5) to block Peyer's, cecal, and colonic patch development in the progeny and induce the development of higher numbers of ILF (12, 14, 15, 25). Some pregnant mice were injected i.v. with 100 μg human IgG (hu-IgG) as a control. The formation of ILF, Peyer's patches, and their patch-like counterparts in the LI is LTβR dependent. However, unlike Peyer's patches and their patch-like counterparts in the LI, ILF formation occurs postnatally. Thus, although *in utero* LTβR-signaling blockade prevents the development of Peyer's, cecal, and colonic patches, the postnatal development of ILF from cryptopatches throughout the SI and LI is conserved (12, 14, 15, 25).

### Prion exposure and disease monitoring.

For oral exposure, mice were fed individual food pellets doused with 50 μl of a 1.0% (wt/vol) dilution of scrapie brain homogenate prepared from mice terminally affected with ME7 scrapie prions (containing approximately 2.5 × 10^4^ intracerebral [i.c.] 50% infective dose [ID_50_] units) according to our standard protocol (3, 5, 26, 27). To do so, during the dosing period mice were individually housed in bedding- and food-free cages. Water was provided ad libitum. A single prion-dosed food pellet was then placed in the cage. The mice were returned to their original cages (with bedding and food ad libitum) as soon as the food pellet was observed to have been completely ingested. The use of bedding-free and additional food-free cages ensured easy monitoring of consumption of the prion-contaminated food pellet. Following prion exposure, mice were coded and assessed weekly for signs of clinical disease and culled at a standard clinical endpoint. The clinical endpoint of disease was determined by rating the severity of clinical signs of prion disease exhibited by the mice. Following clinical assessment, mice were scored as “unaffected,” “possibly affected,” and “definitely affected” using standard criteria that typically present in mice clinically affected with ME7 scrapie prions. Clinical signs following infection with the ME7 scrapie agent may include weight loss, starry coat, hunched posture, jumpy behavior (at early onset) progressing to limited movement, upright tail, wet genitals, decreased awareness, discharge from eyes/blinking eyes, and ataxia of hind legs. The clinical endpoint of disease was defined in one of the following ways: (i) the day on which a mouse received a second consecutive “definite” rating; (ii) the day on which a mouse received a third “definite” rating within four consecutive weeks; (iii) the day on which a mouse was culled in extremis. Survival times were recorded for mice that did not develop clinical signs of disease and were culled when they showed signs of intercurrent disease. Prion diagnosis was confirmed by histopathological assessment of vacuolation in the brain. For the construction of lesion profiles, vacuolar changes were scored in nine distinct gray matter and three distinct white matter areas of the brain as described previously (28).

### Oral T. muris infection.

Trichuris muris was maintained as described previously (29). Mice were infected orally by gavage with ∼200 infective eggs. Some mice were killed at 14 days postinfection, and the worm burden (164 ± 22; *n* = 4) was assessed as described previously (30).

### Immunohistochemisty (IHC) and immunofluorescent analyses.

Whole-mount immunostaining was performed as previously described (16). Briefly, ∼4-cm pieces of intestine were washed in phosphate-buffered saline (PBS) prior to incubation in Hanks' balanced salt solution (HBSS) containing 5 mM EDTA (both from Life Technologies, Paisley, United Kingdom) in a shaking incubator at 37°C. The epithelium was subsequently washed off, and the intestinal pieces were fixed in 10% formal saline (Cellpath, Powys, United Kingdom), washed in Tris-buffered saline containing 0.1% Triton X-100 (Sigma, Poole, United Kingdom) (TBST), and nonspecific binding blocked with 2.5% normal goat serum (Jackson ImmunoResearch, Newmarket, United Kingdom). Intestinal pieces were then stained with rat anti-mouse CD35 monoclonal antibody (MAb; clone 8C12; BD Biosciences) to detect FDC and rat anti-mouse B220 MAb (clone RA3-6B2; Life Technologies) to detect B cells.

Portions of intestine were also removed and snap-frozen at the temperature of liquid nitrogen. Serial frozen sections (10 μm in thickness) were cut on a cryostat and immunostained with antibodies as follows: FDC were visualized by staining with anti-CD35 MAb; cellular PrP^C^ was detected using PrP-specific polyclonal antibody (pAb) 1B3 (31); B cells were detected using rat anti-mouse B220 MAb; M cells were detected using rat anti-mouse GP2 MAb (MBL International, Woburn, MA); mononuclear phagocytes were detected using rat anti-mouse CD11b antibody (clone M1/70; eBioscience, Hatfield, United Kingdom). Nerve synapses were detected using rabbit anti-synaptophysin 1 (Synaptic Systems, Göttingen, Germany). When appropriate, sections were counterstained with 4′,6-diamidino-2-phenylindole (DAPI; Life Technologies).

For the detection of disease-specific PrP (PrP^d^) in intestines, MLN, spleens, and brains, tissues were fixed in periodate-lysine-paraformaldehyde fixative and embedded in paraffin wax. Sections (thickness, 6 μm) were deparaffinized and pretreated to enhance the detection of PrP^d^ by hydrated autoclaving (15 min, 121°C, hydration) and subsequent immersion in formic acid (98%) for 5 min. Sections were then immunostained with 1B3 PrP-specific pAb. For the detection of astrocytes, brain sections were immunostained with anti-glial fibrillary acidic protein (GFAP; Dako, Ely, United Kingdom). For the detection of microglia, deparaffinized brain sections were first pretreated with Target Retrieval Solution (Dako) and subsequently immunostained with anti-ionized calcium-binding adaptor molecule 1 (Iba-1; Wako Chemicals GmbH, Neuss, Germany). For the detection of FDC in intestines, MLN, and spleens, deparaffinized sections were first pretreated with Target Retrieval Solution (Dako) and subsequently immunostained with anti-CD21/35 (clone 7G6; BD Biosciences). Paraffin-embedded tissue (PET) immunoblot analysis was used to confirm the PrP^d^ detected by immunohistochemistry with proteinase K (PK)-resistant PrP^Sc^ (32). Membranes were subsequently immunostained with 1B3 PrP-specific pAb.

For light microscopy, following the addition of primary antibodies, biotin-conjugated species-specific secondary antibodies (Stratech, Soham, United Kingdom) were applied, and immunolabeling was revealed using horseradish peroxidase (HRP) conjugated to the avidin-biotin complex (ABC kit; Vector Laboratories, Peterborough, United Kingdom) and visualized with 3,3′-diaminobenzidine (DAB; Sigma). Sections were counterstained with hematoxylin to distinguish cell nuclei. For fluorescence microscopy, following the addition of primary antibody, streptavidin-conjugated or species-specific secondary antibodies coupled to Alexa Fluor 488 (green), Alexa Fluor 594 (red), or Alexa Fluor 647 (blue) dyes (Life Technologies) were used. Sections were counterstained with either DAPI or Alexa Fluor 647-conjugated phalloidin (Life Technologies) and subsequently mounted in fluorescent mounting medium (Dako).

Whole-mount immunostained intestinal pieces were visualized on a Nikon EC1 confocal microscope (Nikon, Kingston upon Thames, United Kingdom). ILF and mature ILF were enumerated visually along the entire length of the intestinal piece. Images of cryosections were obtained using a Zeiss LSM7 confocal microscope (Zeiss, Welwyn Garden City, United Kingdom).

### Oral gavage with fluorescent microbeads.

Mice were given a single oral gavage of 2 × 10^11^ Fluoresbrite Yellow Green-labeled 200-nm microbeads (Polysciences, Eppelheim, Germany) in 200 μl PBS. Mice were culled 24 h later, and Peyer's patches, SI segments, cecum, and colon were snap-frozen at the temperature of liquid nitrogen. Serial frozen sections (10 μm in thickness) were cut on a cryostat and counterstained with DAPI. Images of follicles from three Peyer's patches per mouse (*n* = 4 mice), cecal patches (1 patch per mouse, *n* = 3 mice), and colonic patches (1 or 2 patches per mouse, *n* = 3 mice) from 4 nonsequential sections (at least 100 μm apart) were acquired using a Nikon Eclipse E400 fluorescence microscope using Micro Manager (http://www.micro-manager.org). Images were acquired for every ILF in nonsequential sections (at least 100 μm apart) of SI (16 sections per mouse, *n* = 4 mice), cecum (4 sections per mouse, *n* = 4 mice), and colon (8 sections per mouse, *n* = 4 mice). The number of beads and the area of lymphoid tissue in each section were determined using ImageJ (http://imagej.nih.gov/ij), and the bead density was calculated. Tissue autofluorescence was subtracted from displayed images using ImageJ.

### Statistical analyses.

Data are presented as means ± standard errors (SE). Unless indicated otherwise, significant differences between samples in different groups were sought by Student's *t* test. In instances where there was evidence of nonnormality, data were analyzed by nonparametric analysis of variance (ANOVA) (Kruskal-Wallis test) with Dunn's multiple comparison *post hoc* test. *P* values of <0.05 were accepted as significant.

## RESULTS

### Mice with FDC-containing GALT predominantly in the large intestine.

**FIG 1:**
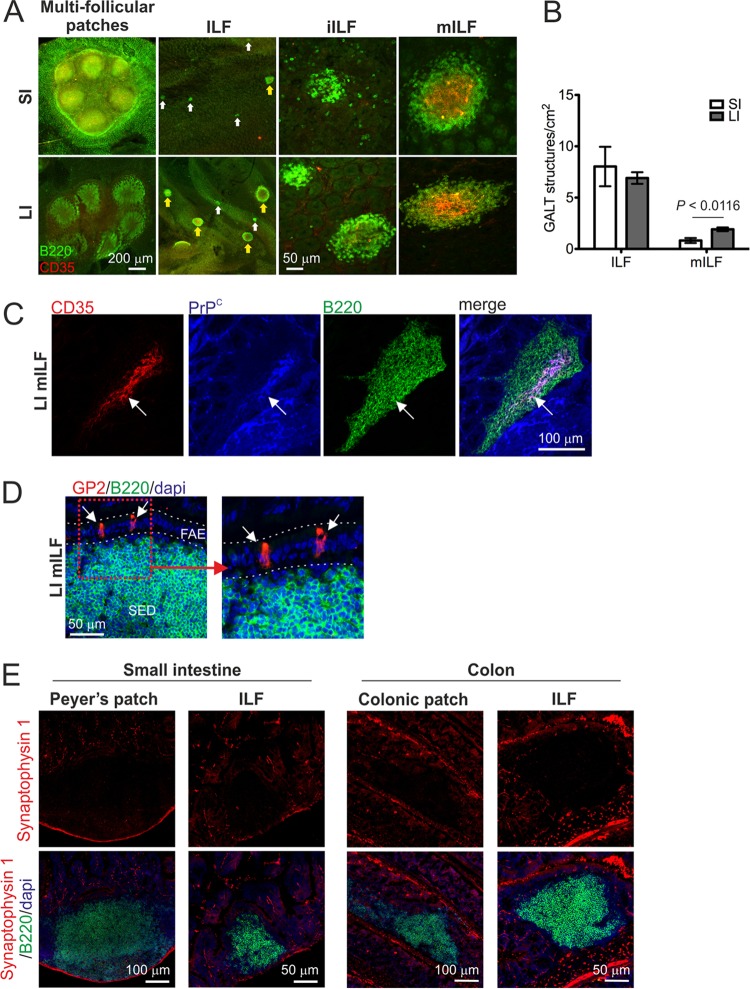
GALT status in the small and large intestines. (A) Whole-mount IHC analysis of the GALT in the intestines of adult C57BL/6J mice. Intestinal pieces were whole-mount immunostained to detect B-cell follicles (CD45R/B220^+^ cells; green) and FDC networks (CD35^+^ cells; red). In the small intestine (SI) the ILF were mostly immature (iILF) and lacked FDC networks (white arrows). In the large intestine (LI), many of the ILF were mature (mILF) and contained FDC networks (yellow arrows). (B) Determination of ILF and mILF density in the SI and LI of adult C57BL/6J mice (open bars and closed bars, respectively). Data are derived from the whole intestines and are presented as the mean number of GALT structures/cm^2^ (*n* = 4 mice/group). (C) IHC detection of PrP^C^-expressing FDC networks in LI mILF. Cryosections of colon were immunostained to detect FDC (CD35^+^ cells; red), PrP^C^ (blue), and B cells (CD45R/B220; green). (D) The follicle-associated epithelia (FAE) overlying ILF in the LI contain glycoprotein 2-expressing mature M cells (GP2; red). Cryosections were counterstained to detect B cells (CD45R/B220; green) and cell nuclei (DAPI; blue). The boxed area in the left-hand panel is presented at higher magnification in the right-hand panel. (E) Comparison of the innervation associated with the GALT in the SI and LI. Sections of intestines were immunostained to detect nerve synapses (synaptophysin 1; red), B-cell follicles (CD45R/B220^+^ cells; green), and cell nuclei (DAPI; blue).

To study the relative contributions of the GALT in the SI and LI to oral prion disease pathogenesis, we first created mice in which the FDC-containing GALT was found predominantly in the LI at the time of exposure. Initially, the GALT in the SI and LI of adult C57BL/6J mice were characterized by whole-mount immunostaining of entire intestines to detect the presence of B-cell follicles (CD45R/B220^+^ cells; green) and FDC networks (CD35^+^ cells; red) (16). The aim here was to determine the status of the LI GALT and whether it was potentially capable of supporting prion uptake and accumulation. The SI typically contained 5 to 7 multifollicular Peyer's patches and numerous isolated lymphoid follicles (ILF) (Fig. 1A). ILF can be classified as either immature ILF (primary B-cell follicles) or mature ILF containing a single organized B-cell-containing germinal center, an FDC network, and an overlying M-cell-containing follicle-associated epithelium (FAE) (12–16, 33). In the SI of adult C57BL/6J mice, the ILF were almost entirely immature and lacked FDC networks (Fig. 1A and B). In the LI, a number of multifollicular patch-like structures and ILF were also identified (Fig. 1A). However, a significantly higher number of the ILF within the LI were mature and contained FDC networks than within the SI (Fig. 1A and B; *P* < 0.0116).

The transcytosis of prions across the intestinal epithelium by M cells and their subsequent replication upon PrP^C^-expressing FDC are obligatory for efficient neuroinvasion after oral exposure (3, 5, 27, 34). Immunohistochemical (IHC) analysis confirmed that the mature ILF in the LI contained PrP^C^-expressing FDC networks (Fig. 1C, arrow) and glycoprotein 2-expressing mature M cells within the overlying epithelium (Fig. 1D, arrows) (35). Furthermore, the density and distribution of the enteric innervation associated with the GALT in the SI and LI appeared to be similar (Fig. 1E) (36). These data suggest that the GALT in the LI also have the potential to be important sites of prion accumulation and neuroinvasion.

The multifollicular Peyer's patches in the SI and their counterparts in the LI (cecal and colonic patches) are dependent on lymphotoxin β receptor (LTβR) signaling during embryogenesis for formation and are absent in LT-deficient mice (37) or mice treated with LTβR-Ig *in utero* (25). ILF formation is also LTβR dependent (3, 12, 14, 16). However, unlike Peyer's patches and their patch-like counterparts in the LI, ILF formation occurs postnatally and their development from cryptopatches in the intestines of LT-deficient mice can be induced by reconstitution with LT-expressing (wild-type) hematopoietic cells (3, 12–14, 16, 38). Although *in utero* an LTβR-signaling blockade prevents the development of Peyer's, cecal, and colonic patches, the postnatal development of ILF throughout the SI and LI is conserved, with higher numbers of ILF observed due to the absence of other GALT (14, 15).

**FIG 2:**
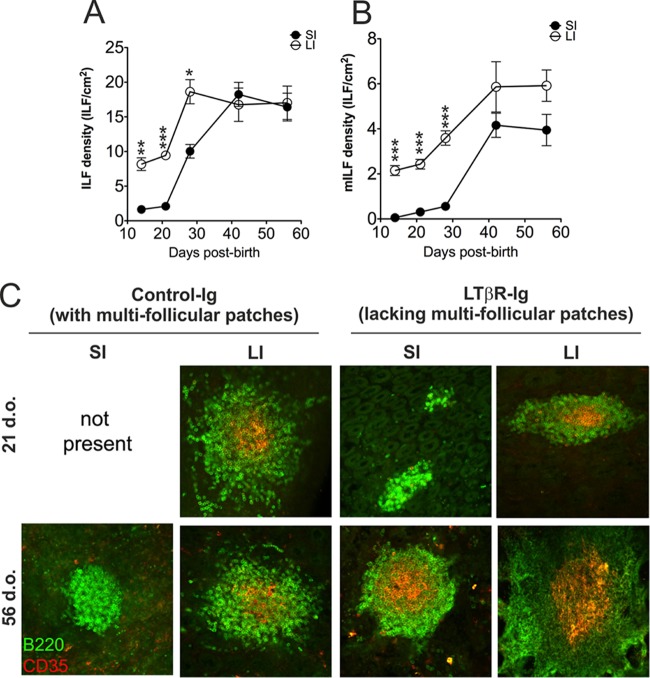
GALT status in the small and large intestines of *in utero* LTβR-Ig-treated mice. (A, B) C57BL/6J mice were treated *in utero* with LTβR-Ig on day E11.5 to block Peyer's, cecal, and colonic patch development and induce the development of higher numbers of ILF. At intervals after birth, entire intestines were whole-mount immunostained to detect B-cell follicles and FDC networks. Mice were culled at intervals after birth, and the total ILF (A) and mILF (B) in the SI and LI (closed and open symbols, respectively) were counted; *n* = 4 mice/group; *, *P* < 0.01; **, *P* < 0.001; ***, *P* < 0.0001. (C) Whole-mount immunostaining of ILF status (CD45R/B220^+^ cells, green; CD35^+^ cells, red) in the intestines of 21-day-old (upper panels) and 56-day-old (lower panels) control Ig- and LTβR-Ig-treated mice (*n* = 4 mice/group).

The postnatal formation and maturation of ILF in the LI occurs at a significantly earlier time after birth than in the SI (15). Therefore, by exploiting these differing developmental kinetics, mice with FDC-containing GALT predominantly in the LI could be generated. ILF development in the SI and that in the LI of *in utero* LTβR-Ig-treated mice (termed “LTβR-Ig-treated mice” hereinafter) were compared to establish the optimal time when FDC-containing GALT (mature ILF) were present only in the LI. Pregnant C57BL/6J mice were injected i.v. with LTβR-Ig (24) (or hu-IgG as a control) on day E11.5 to block Peyer's, cecal, and colonic patch development in the progeny and induce the development of higher numbers of ILF (12, 14, 15, 25). At intervals after birth, entire intestines were whole-mount immunostained to detect B-cell follicles (CD45R/B220^+^ cells; green) and FDC networks (CD35^+^ cells; red) (16). Our analysis showed that the LI of 21-day-old LITβR-Ig-treated mice contained significantly more ILF than the SI (Fig. 2A). Furthermore, many of these ILF were mature and contained FDC networks (Fig. 2B). In the SI of LTβR-Ig-treated mice at this time, few if any mature ILF were detected (Fig. 2B). These data clearly show that in the intestines of 21-day-old LTβR-Ig-treated mice the predominant FDC-containing GALT were the mature ILF in the LI (Fig. 2C) (hereinafter termed “mice with FDC-containing GALT only in the LI”). In contrast, by 56 days after birth, many FDC-containing mature ILF were distributed throughout the SI and LI (termed “mice with FDC-containing GALT throughout the SI and LI” hereinafter). Thus, by exposing LTβR-Ig-treated mice to prions at 21 or 56 days after birth, we could determine whether FDC-containing GALT in the SI or the LI were important sites of prion accumulation and neuroinvasion after oral exposure.

### The GALT in the LI are not early sites of prion accumulation after oral exposure.

Next, we determined where the important sites of prion accumulation were after oral exposure. If the SI was the major site, we hypothesized that the specific absence of FDC-containing GALT in the SI at the time of exposure (LTβR-Ig-treated mice exposed to prions at 21 days after birth) would block neuroinvasion from the intestine. Additionally, disease pathogenesis would be unaffected in LTβR-Ig-treated mice exposed to prions at 56 days after birth (which contain mature ILF in the SI and LI) compared to controls. Conversely, if the LI played an important role, prion pathogenesis would be unaffected in mice with FDC-containing GALT restricted to the LI at the time of exposure (LTβR-Ig-treated mice exposed to prions at 21 days after birth).

Pregnant C57BL/6J mice were injected i.v. with LTβR-Ig (or hu-IgG as a control) on day E11.5 to block Peyer's, cecal, and colonic patch development in the progeny and induce the development of higher numbers of mature ILF. At either 21 days (LI FDC-containing GALT only) or 56 days (SI and LI FDC-containing GALT) after birth, mice were orally exposed to ME7 scrapie prions. The GALT status in the intestines of each treatment and control group used in this study at the time of oral prion exposure is described in Table 1. Both PET immunoblot (32) and IHC were used to detect disease-specific PrP accumulations characteristically found only in prion-affected tissues and considered a reliable biochemical marker for the presence of infectious prions (3, 5, 26, 27). PET immunoblot analysis detects prion disease-specific, relatively proteinase K (PK)-resistant PrP^Sc^. However, as PK destroys tissue microarchitecture, disease-specific abnormal accumulations of PrP (PrP^d^) were detected by IHC (3, 27, 34).

**TABLE 1:** GALT status in the intestines of each experimental group at the time of oral prion exposure

Treatment^*a*^	Age of mice (days)	Small intestine GALT status			Large intestine GALT status		
		Peyer's patches	Density of immature ILF^*b*^	Density of mature ILF	Cecal and colonic patches	Density of immature ILF	Density of mature ILF
Control IgG	21	Present	0 ± 0	0 ± 0	Present	2 ± 0	1 ± 0
LTβR-Ig	21	Absent	1 ± 0	0 ± 0	Absent	3 ± 0	2 ± 0
Control IgG	56	Present	5 ± 1	0 ± 0	Present	3 ± 0	1 ± 0
LTβR-Ig	56	Absent	17 ± 4	5 ± 1	Absent	6 ± 1	3 ± 0

a Pregnant mice were injected i.v. with LTβR-Ig on day E11.5 or with control IgG. Progeny mice were analyzed at the ages indicated.

b Entire SI and LI were whole-mount immunostained to detect the presence of B-cell follicles (CD45R/B220^+^ cells) and FDC networks (CD35^+^ cells), and the number and status of ILF were recorded. Density values are mean numbers of ILF/cm^2^ ± SE; *n* = 4 mice/group.

**FIG 3:**
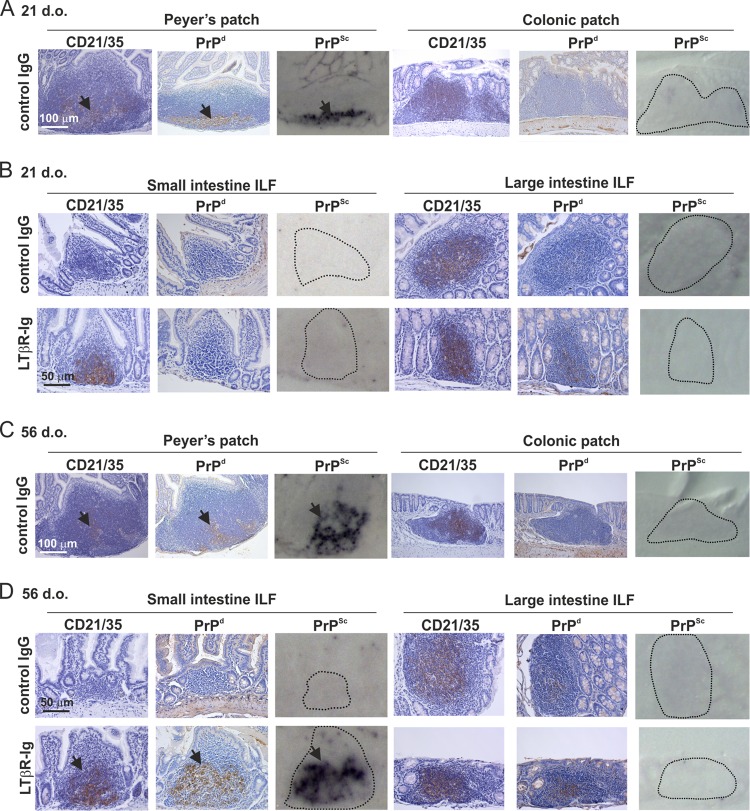
FDC-containing GALT in the large intestine are not early sites of prion accumulation after oral exposure. C57BL/6J mice were treated *in utero* with LTβR-Ig on day E11.5 to block Peyer's, cecal, and colonic patch development and induce the development of higher numbers of ILF. Control mice were treated with hu-Ig. At 21 (A and B) or 56 (C and D) days old (d.o.), mice were orally exposed to ME7 scrapie prions, and entire intestines were collected 105 days after exposure. (A and C) High levels of PrP^d^ (brown) were detected in association with FDC (CD21/35-positive cells; brown) in the Peyer's patches in the SI of control mice (arrows). Analysis of adjacent sections by PET immunoblot analysis confirmed the presence of PK-resistant PrP^Sc^ (blue/black). In contrast, no PrP^d^ or PrP^Sc^ was detected the in colonic patches in the LI of the same control mice. (B) In mice with FDC-containing GALT (mature ILF) only in the LI at the time of oral prion exposure (21-d.o. LTβR-Ig-treated mice), PrP^d^/PrP^Sc^ accumulation in the GALT was blocked. (D) In contrast, in mice with FDC-containing GALT (mature ILF) throughout the SI and LI at the time of oral prion exposure (56-d.o. LTβR-Ig-treated mice), high levels of PrP^d^ and PrP^Sc^ were detected in association with FDC (CD21/35-positive cells) in the mature ILF in the SI (arrows) but were undetectable in the LI. Sections were counterstained with hematoxylin to detect cell nuclei (blue). For all panels, there were 4 mice/group.

In the SI of all control IgG-treated mice, heavy PrP^d^ accumulations were detected in the Peyer's patches at 15 weeks after oral prion exposure, consistent with localization upon CD21/35-expressing FDC (Fig. 3A and C, arrows). PET immunoblot of adjacent sections confirmed the presence of high levels of prion-specific PrP^Sc^ in Peyer's patches from control IgG-treated mice (Fig. 3A and C). However, PrP^d^ was undetectable in colonic patches (Fig. 3A and C) or in immature ILF throughout the SI and the immature ILF and occasional mature ILF in the LI (Fig. 3B and D, upper panels). In mice with only LI FDC-containing GALT at the time of exposure (LTβR-Ig-treated mice exposed to prions at 21 days after birth), no PrP^Sc^ was detected in the mature ILF in the LI or the ILF in the SI (Fig. 3B, lower panels). However, in the intestines of mice with abundant FDC-containing mature ILF throughout the SI and LI at the time of oral prion exposure (LTβR-Ig-treated mice exposed to prions at 56 days after birth), heavy PrP^Sc^ accumulations were detected in the mature ILF in the SI but not in those in the LI (Fig. 3D, lower panels).

**FIG 4:**
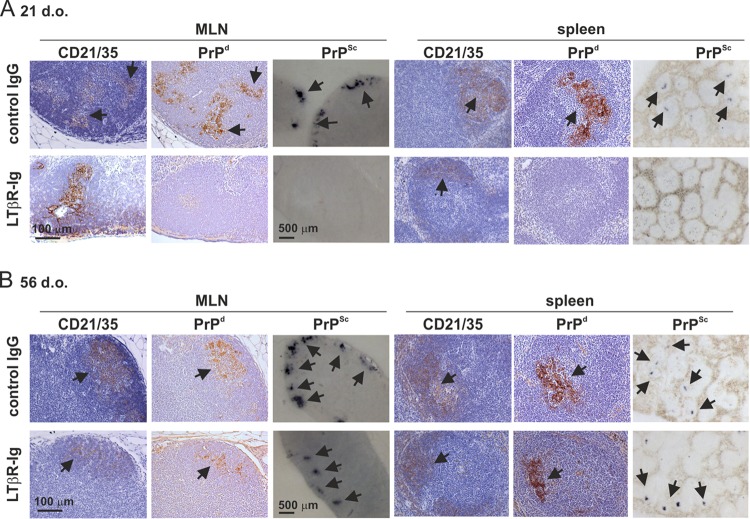
In the absence of FDC-containing GALT in the SI at the time of oral prion exposure, the accumulation of PrP^Sc^ in the MLN and spleen is impeded. C57BL/6J mice were treated *in utero* with LTβR-Ig on day E11.5 to block Peyer's, cecal, and colonic patch development and induce the development of higher numbers of ILF. Control mice were also treated with hu-Ig. At 21 (A) or 56 (B) days old (d.o.), mice were orally exposed to ME7 scrapie prions, and the mesenteric lymph nodes (MLN) and spleen were collected 105 days after exposure. (A) High levels of PrP^d^ were detected in association with FDC (CD21/3-positive cells; brown) in the MLN and spleens of control mice (upper panels; arrows). Analysis of adjacent sections by PET immunoblot analysis confirmed the presence of PK-resistant PrP^Sc^ (blue-black; arrows). In contrast, in the absence of FDC-containing GALT in the SI at the time of oral prion exposure, the accumulation of PrP^d^ and PrP^Sc^ in the in MLN and spleen was blocked (lower panels). (B) However, in mice with FDC-containing GALT throughout the SI and LI at the time of oral prion exposure, high levels of PrP^d^ and PrP^Sc^ were detected in association with FDC in the MLN and spleen (lower panels; arrows). Sections were counterstained with hematoxylin to detect cell nuclei (blue). For all panels, there were 4 mice/group.

After oral exposure, prions first accumulate in the GALT before spreading to other lymphoid tissues, including the MLN and spleen (3, 5, 26). Here, by 15 weeks after oral prion exposure, heavy PrP^Sc^ accumulations were also detectable upon FDC in the MLN and spleen of control IgG-treated mice (Fig. 4A and B, upper panels) (26, 27). In contrast, in mice with FDC-containing GALT only in the LI at the time of exposure (LTβR-Ig-treated mice exposed to prions at 21 days after birth), the subsequent spread of prions to the MLN and spleen was impeded (Fig. 4A, lower panels). However, high levels of PrP^Sc^ were detected upon FDC in the MLN and spleen of mice with FDC-containing mature ILF throughout the SI and LI at the time of oral exposure (LTβR-Ig-treated mice exposed to prions at 56 days after birth) (Fig. 4B, lower panels).

These data clearly show that the GALT in the SI are the major early sites of prion accumulation after oral exposure. Furthermore, in the specific absence of FDC-containing GALT in the SI, the subsequent dissemination of prions from the GALT to other lymphoid tissues is impeded.

### The GALT in the SI, not the LI, are important sites of prion neuroinvasion after oral exposure.

**FIG 5:**
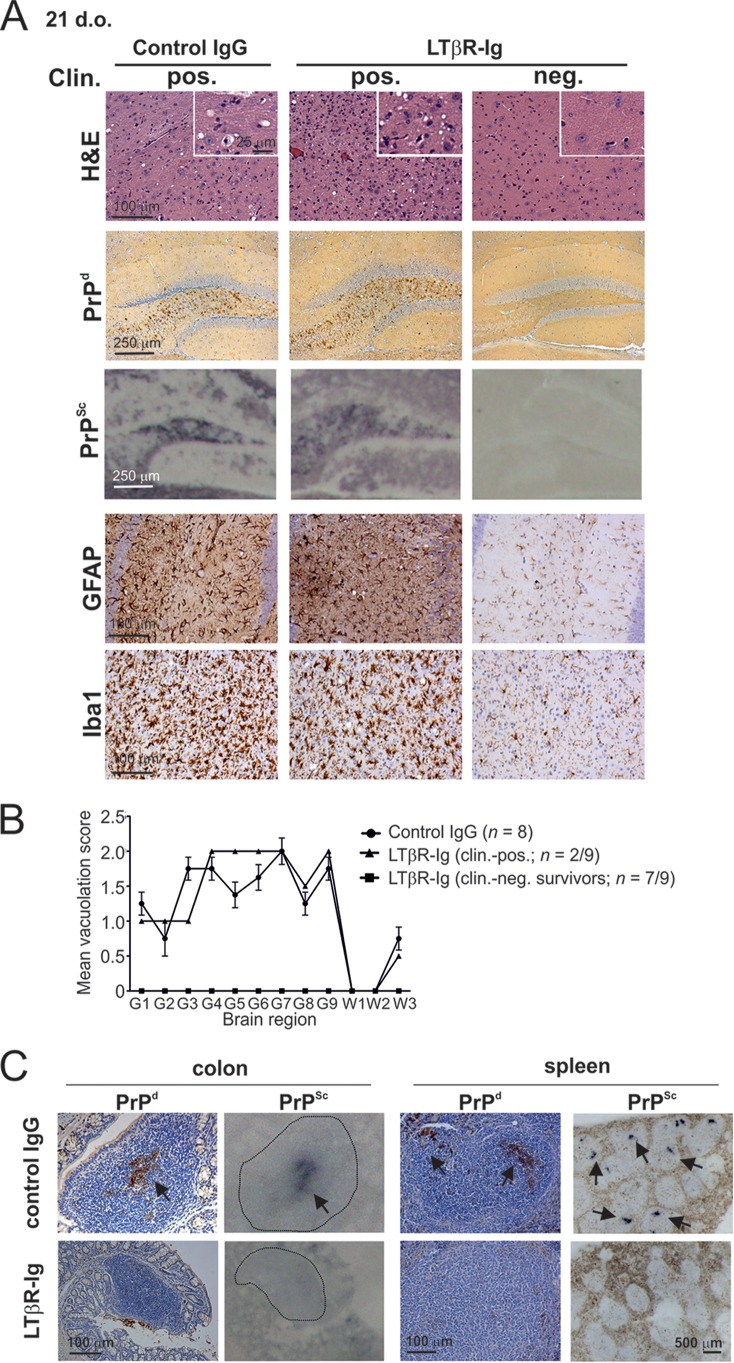
Prion neuroinvasion is impeded in the absence of FDC-containing GALT in the SI at the time of oral exposure. (A) High levels of spongiform pathology (H&E, upper row), heavy accumulations of PrP^d^ (brown, second row) and disease-specific PrP^Sc^ (blue-black, third row), reactive astrocytes expressing GFAP (brown, third row) and active microglia expressing Iba-1 (brown, bottom row) were detected in the brains of all orally exposed clinically scrapie-affected control IgG-treated mice (left-hand panels; *n* = 8). However, most of the mice with FDC-containing GALT only in the LI at the time of exposure (LTβR-Ig-treated mice exposed to prions at 21 days old, right-hand panels, *n* = 7/9) remained free of the clinical and histopathological signs of prion disease up to at least 518 days after oral exposure. Clin., clinical prion disease status; pos., positive; neg., negative. The insets in the upper H&E panels show a representative area from the same image at higher magnification. (B) The severity and distribution of the spongiform pathology (vacuolation) within each brain were scored on a scale of 1 to 5 in nine gray matter and three white matter areas: G1, dorsal medulla; G2, cerebellar cortex; G3, superior colliculus; G4, hypothalamus; G5, thalamus; G6, hippocampus; G7, septum; G8, retrosplenial and adjacent motor cortex; G9, cingulate and adjacent motor cortex; W1, inferior and middle cerebellar peduncles; W2, decussation of superior cerebellar peduncles; and W3, cerebellar peduncles. Each point represents the mean vacuolation score ± SE. (C) At the clinical stage of disease, high levels of PrP^d^ (brown) were detected in association with FDC in the ILF in the LI and the spleens of control mice (upper panels; arrows). Analysis of adjacent sections by PET immunoblot analysis confirmed the presence of PK-resistant PrP^Sc^ (blue-black; arrows). In contrast, in the absence of FDC-containing GALT in the SI at the time of oral prion exposure, the accumulation of PrP^d^ and PrP^Sc^ in the GALT in the LI and spleen was blocked (lower panels). Sections were counterstained with hematoxylin to detect cell nuclei (blue). Data are representative of tissues from 8 or 9 mice/group.

Efficient neuroinvasion following oral exposure of mice to prions is dependent upon FDC-containing GALT (3–5), but whether this occurs via the SI or LI GALT is uncertain. We next compared the influence of SI and LI GALT on neuroinvasion and disease susceptibility. Consistent with the high levels of early PrP^Sc^ accumulation upon FDC within the GALT (Fig. 3C and D), mice with mature ILF throughout the SI and LI at the time of oral exposure (56-day-old LTβR-Ig-treated mice) succumbed to clinical prion disease at the same time as control mice (322 ± 2 and 321 ± 7 days, respectively; *n* = 6/group). However, whereas all control mice orally exposed to prions at 21 days old succumbed to clinical prion disease, those with LI FDC-containing GALT only at the time of exposure displayed dramatically reduced disease susceptibility (21-day-old LTβR-Ig-treated mice). Seven of nine of these mice remained free of clinical signs of prion disease ≥518 days after oral exposure, at which time the experiment was terminated (Table 2). Characteristic spongiform pathology, astrogliosis, microgliosis, and PrP^Sc^ accumulation typically associated with terminal infection with ME7 scrapie prions were detected in the brains of all clinically affected mice (Fig. 5A and B). In contrast, no histopathological signs of prion disease were detected within the brains of any of the clinically negative mice (Fig. 5A and B).

**TABLE 2:** Influence of the large intestine GALT on oral prion disease susceptibility

Treatment^*a*^	Presence of FDC in GALT at time of exposure^*b*^	No. of animals showing PrP^Sc^ accumulation in GALT/total no.	Mean incubation period (days ± SE)^*c*^	Incidence of:	
				Clinical disease^*d*^	Histopathological signs of prion disease in the brain^*e*^
Control IgG	PP, SI-ILF, CP, LI-ILF	8/8	387 ± 17	8/8	8/8
LTβR-Ig	LI-ILF only	2/9	**323, 344,** 7 X > 518	2/9	2/9

a Pregnant mice were injected i.v. with LTβR-Ig or control IgG, and the progeny mice were orally exposed to ME7 scrapie prions when 21 days old.

b PP, Peyer's patches; SI-ILF, small intestine isolated lymphoid follicles; CP, cecal and colonic patches; LI-ILF, large intestine isolated lymphoid follicles.

c The notation “*n* X > 518” means that mice were free of the clinical and pathological signs of prion disease up to at least this duration after oral exposure. Boldface values represent individual incubation periods for individual clinically and pathologically prion disease-positive mice.

d No. of animals displaying clinical signs of prion disease/no. of animals tested.

e No. of animals with histopathological signs of prion disease in the brain (vacuolation in the neuropil and PrP^Sc^ accumulation)/no. of animals tested.

At the terminal stage of disease, high levels of PrP^Sc^ were maintained upon FDC in the SI GALT and spleen of all control mice (Fig. 5C). Furthermore, at the terminal stage of disease in control-treated mice, heavy PrP^Sc^ accumulations were now also detected upon FDC within the ILF in the LI (Fig. 5C, upper left-hand panels, arrows). However, in mice with FDC-containing GALT only in the LI at the time of prion exposure, no evidence of PrP^Sc^ accumulation within their GALT and spleens was observed (Fig. 5C, lower panels), implying that disease pathogenesis had been impeded. These data clearly show that in the specific absence of FDC-containing GALT in the SI, prion neuroinvasion following oral exposure is substantially impaired, demonstrating that SI FDC-containing GALT are the important early sites of prion accumulation or neuroinvasion after oral exposure.

### Effect of congruent Trichuris muris infection on oral prion pathogenesis.

**FIG 6:**
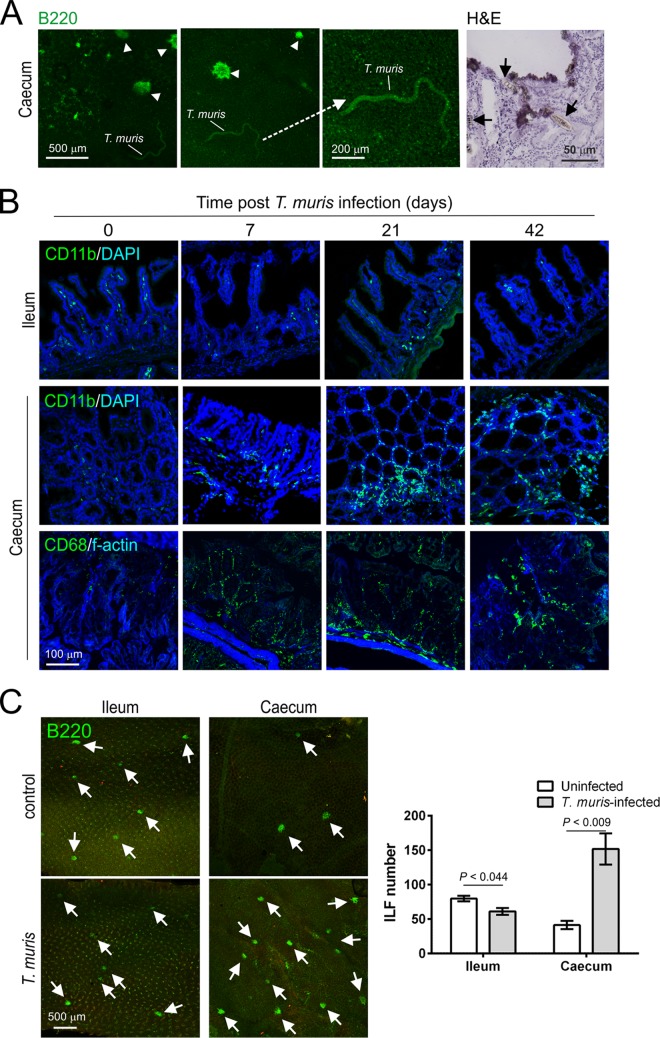
Infection with the nematode parasite Trichuris muris is restricted to the cecum. Groups of mice were orally infected with ∼200 T. muris infective eggs, and tissues were collected at intervals after exposure. (A) T. muris establishes infection in the cecal epithelium. Left-hand panels show autofluorescent immature worms adhered to the cecal epithelium. Clusters of B-cell follicles (ILF, CD45R/B220^+^ cells, green) are indicated (arrowheads). The right-hand panel (H&E) shows the close association of T. muris with the cecal epithelium/lamina propria (arrows) and sites of damage to the gut epithelium. Tissues were analyzed on day 14 (left-hand and middle panels) and day 7 (right-hand panel) postinfection with T. muris. (B) T. muris infection stimulates the influx of macrophages (CD11b^+^ cells, green, upper and middle panels; CD68^+^ cells, green, lower panels) into the lamina propria of the cecum, but not in the SI. (C) The distal 8 cm of ileum and the entire cecum were whole-mount immunostained to detect B cell follicles (B220^+^ cells; green). T. muris infection stimulates the development of abundant ILF (arrows) in cecum but not the SI. The histogram shows that the number of ILF in the cecum of T. muris-infected mice was significantly greater than that observed in controls. Tissues were analyzed on day 28 postinfection with T. muris. Data in all panels are derived from analysis of tissues from 4 mice/group.

We next determined whether pathology restricted to the LI may influence oral prion disease pathogenesis. For example, pathology to the LI mucosa may enhance disease pathogenesis by increasing prion uptake across the intestinal epithelium. Conversely, it is plausible that lymphocytes and macrophages infiltrating the lamina propria may decrease susceptibility due to prion sequestration (34, 39). These pathological characteristics are observed in the LI during murine Trichuris muris infection, a well-characterized natural mouse model of T. trichiura, one of the most prevalent human helminth infections worldwide. T. muris infection is restricted to the LI, where it burrows within the epithelium (29) (Fig. 6A). Peak expulsion coincides with the influx of large numbers of macrophages (CD11b^+^ and CD68^+^ cells) into the lamina propria of the LI (40) (Fig. 6B). T. muris infection also stimulates the development of ILF in the LI (40) (Fig. 6C). This parasite has distinct advantages for use in this study as the infection does not affect the SI (Fig. 6). T. muris is also a natural mouse pathogen and does not require antibiotic treatment or fasting to establish infection, which may influence oral prion disease pathogenesis.

Groups of mice were orally infected with ∼200 T. muris infective eggs and subsequently orally exposed to ME7 scrapie prions at the following specific intervals after T. muris infection to determine whether the parasite-induced pathology in the LI may influence prion disease pathogenesis: (i) day 0, mice were exposed to T. muris and prions at the same time; (ii) day 7, T. muris infection was established in the LI, coincident with the intracellular presence of the first larval stage of the parasite within an epithelial syncytium (Fig. 6A); (iii) day 21, time of peak T. muris clearance, coincident with the influx of macrophages into the lamina propria (Fig. 6B), and the subsequent significant increase in the number of ILF in the LI by day 28 (40, 41) (Fig. 6C); (iv) day 42, approximately 7 days after clearance of T. muris. An additional group of mice were orally exposed to ME7 scrapie prions alone as a control.

**FIG 7:**
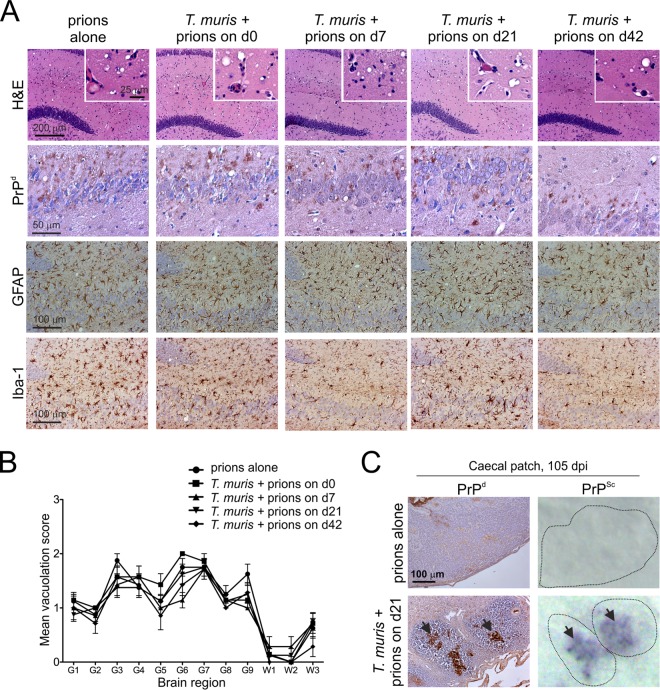
Congruent T. muris infection did not influence the severity and distribution of the histopathological signs of prion disease in the brains of clinically affected mice. Groups of mice were orally infected with ∼200 T. muris infective eggs and subsequently orally exposed to prions on the days (d) indicated in relation to the ongoing T. muris infection in the large intestine. (A) Brains were collected from all mice with clinical prion disease, and the neuropathological signs of prion disease were compared. High levels of spongiform pathology (H&E, upper row), heavy accumulations of PrP^d^ (brown, second row), reactive astrocytes expressing GFAP (brown, third row), and active microglia expressing Iba-1 (brown, bottom row) were detected in the brains of all clinically scrapie-affected mice. The insets in the upper H&E panels show a representative area from the same image at higher magnification. (B) The severity and distribution of the spongiform pathology (vacuolation) within each brain were scored on a scale of 1 to 5 in nine gray matter and three white matter areas: G1, dorsal medulla; G2, cerebellar cortex; G3, superior colliculus; G4, hypothalamus; G5, thalamus; G6, hippocampus; G7, septum; G8, retrosplenial and adjacent motor cortex; G9, cingulate and adjacent motor cortex; W1, inferior and middle cerebellar peduncles; W2, decussation of superior cerebellar peduncles; and W3, cerebellar peduncles. Each point represents the mean vacuolation score ± SE. Data are representative of tissues from 7 or 8 mice/group. (C) At 105 days after oral prion infection, no PrP^d^ or PrP^Sc^ was detected the in cecal patches in the LI of mice orally exposed to prions alone (upper panels). In contrast, high levels of PrP^d^ and PrP^Sc^ were detected in the cecal patches of prion-infected mice with congruent T. muris infection (lower panels, arrows). Representative images are from mice exposed to prions at 21 days after T. muris infection. Data are representative of tissues from 4 mice/group.

Irrespective of the time at which mice were coexposed with prions, all mice succumbed to clinical prion disease with incubation periods similar to those of mice exposed to prions alone (Table 3). Congruent T. muris infection also did not influence the severity and distribution of the histopathological signs of prion disease in the brains of any of the clinically affected mice (Fig. 7A and B). However, at 15 weeks after oral prion exposure, high levels of PrP^Sc^ were detectable in the LI of mice with congruent T. muris infection, in contrast to mice exposed to prions alone (Fig. 7C). These data clearly show that pathology specifically restricted to the LI, such as that which occurs during T. muris infection, does not affect the onset or severity of oral prion disease but can facilitate the earlier accumulation of prions within LI GALT.

**TABLE 3:** Effect of congruent Trichuris muris infection on oral prion disease pathogenesis

Mouse treatment	Characteristic T. muris-mediated pathology in LI mucosa	Mean prion disease incubation period (days ± SE)	Incidence of:	
			Clinical disease^*b*^	Histopathological signs of prion disease in the brain^*c*^
Prions alone	None	343 ± 9	8/8	8/8
T. muris infection, followed by prions at day^*a*^:				
0	None	350 ± 7	7/7	7/7
+7	Syncytial tunnels in epithelium	356 ± 12	7/7	7/7
+21	Influx of intraepithelial macrophages	356 ± 13	8/8	8/8
+42	7 days after expulsion of T. muris	347 ± 4	7/7	7/7

a Mice were orally infected with T. muris and then were orally exposed to ME7 scrapie prions after the indicated number of days.

b No. of animals displaying clinical signs of prion disease/no. of animals tested.

c No. of animals with histopathological signs of prion disease in the brain (vacuolation in the neuropil and PrP^Sc^ accumulation)/no. of animals tested.

### Large intestinal GALT is relatively deficient in the uptake of orally administered particulate antigen.

The absence of prion accumulation in the LI GALT at early time points contrasted with that observed at later stages of disease and suggested that the LI GALT does not efficiently uptake orally acquired prions from the gut lumen. While the epithelia covering both the SI and the LI GALT have M cells (Fig. 1D) (27), region-specific differences in factors such as mucus thickness (42) may play a role in preventing prion uptake in the LI. It was unclear whether reduced uptake in the LI was specific to prions or whether the uptake of other orally administered particulate antigens was similarly reduced. Microbeads are commonly used to assess M-cell uptake of particulate antigens and after administration are readily detected within or below M-cell-rich areas overlying GALT, but not within the villous epithelium and the underlying lamina propria (27, 33, 43). We therefore considered these microbeads a good model for prion uptake in the intestine, as both are transported by M cells and lack any means of self-propulsion. Additionally, the use of fluorescently labeled microbeads enables them to be readily tracked histologically, which would not be possible following oral exposure to a physiologically relevant (low) dose of fluorescently labeled prions.

**FIG 8:**
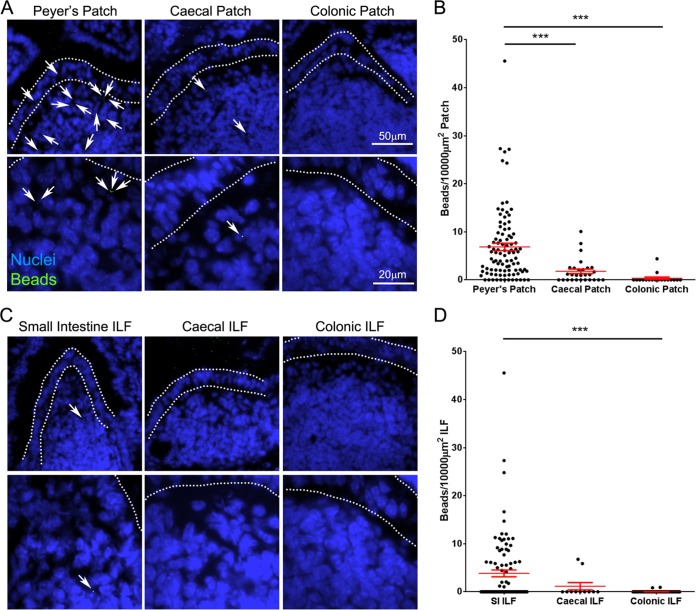
Large intestinal GALT is relatively deficient in the uptake of particulate antigen. Mice were orally gavaged with 2 × 10^11^ 200-nm fluorescent microbeads. At 24 h following gavage, the Peyer's patches, small intestine, cecum, and colon were collected. Cryosections of each were prepared and counterstained with DAPI to detect cell nuclei (blue). (A) Images of Peyer's, cecal, and colonic patches showing microbead (green) accumulation. Microbeads within patches are highlighted with arrows. The follicle-associated epithelium is defined by dotted lines. (B) The number of beads in sections of Peyer's (*n* = 96), cecal (*n* = 28), and colonic (*n* = 18) patches from 3 or 4 mice was determined, and the area of GALT was measured to determine the relative microbead density. Each dot represents the microbead density of an individual patch follicle. Bars display means ± standard errors of the means (SEM). (C) Images of small intestinal, cecal, and colonic isolated lymphoid follicles (ILF) showing microbead (green) accumulation. Microbeads within ILF are highlighted with arrows. The follicle-associated epithelium is defined by dotted lines. (B) The number of beads in small intestinal (*n* = 95), cecal (*n* = 11), and colonic (*n* = 64) ILF from 4 mice was determined, and the area of GALT was measured to determine the relative microbead density. Each dot represents the microbead density of an individual ILF. Bars display means ± SEM. Significant differences were determined by nonparametric ANOVA (Kruskal-Wallis test) with a Dunn's multiple comparison *post hoc* test. ***, *P* < 0.001.

To determine if microbeads were acquired by both SI and LI GALT, C57BL/6J mice (not treated *in utero* with LTβR-Ig) were orally gavaged with 2 × 10^11^ 200-nm fluorescent microbeads, and 24 h later, the presence of microbeads in cryosections of Peyer's, cecal, and colonic patches was analyzed. While microbeads were readily observable in Peyer's patches in the SI, significantly fewer were observed in the cecal patches in LI, despite numerous microbeads in the lumen (Fig. 8A and B). The presence of microbeads within colonic patches was rare. A similar pattern was observed in ILF, with much higher densities of microbeads observed in SI ILF than in those in the LI (Fig. 8C and D), despite the increased ILF maturity (associated with the development of an M-cell-containing FAE) observed in the colon (Fig. 1B and D). Therefore, orally administered, nonmotile particulate antigens, such as prions, are preferentially taken up into SI GALT and rarely acquired by the LI GALT.

## DISCUSSION

Here we show that the SI GALT are the major early sites of prion accumulation and neuroinvasion after oral exposure. In the absence of SI FDC-containing GALT at the time of oral exposure, prions failed to accumulate in the remaining FDC-containing GALT in the LI, dramatically reducing disease susceptibility and rendering the mice refractory to infection. Oral prion disease pathogenesis in natural hosts shows temporal characteristics similar to those observed in the current study (21–23), suggesting that the SI GALT are also the major early sites of prion accumulation and neuroinvasion during natural prion infections. Congruent infection with the LI-restricted pathogen T. muris did facilitate earlier prion accumulation in LI GALT, but ultimately this and the presence of significant LI pathology around the time of prion exposure did not influence prion neuroinvasion, underlining the important role of the SI GALT in this process. Together, these data demonstrate that the FDC-containing GALT in the SI, specifically Peyer's patches and mature ILF, are the major early sites of prion accumulation and neuroinvasion after oral exposure.

Our data show that Peyer's patches and mature ILF in the SI are each individually capable of supporting prion accumulation and neuroinvasion. The only FDC-containing GALT in the SI of the 21-day-old control IgG-treated and 56-day-old LTβR-Ig-treated mice were Peyer's patches and mature ILF, respectively, and both were fully susceptible to oral prion infection. In the absence of FDC-containing GALT in the SI, the gastrointestinal tract appears to act as a barrier against oral prion infection. Unfortunately, it is not currently possible to create mice with FDC-containing GALT exclusively in the SI.

Prions are acquired from the gut lumen via M cells (27, 44, 45), specialized epithelial cells that transcytose luminal antigens (46). Although M cells are abundant in SI Peyer's patches, they are less numerous in cecal patches (47). Our data show that LI GALT are also much less efficient at transcytosing luminal antigens. The LI epithelium is also covered with a thick layer of mucus (42). Both of these factors likely contribute to the inability of LI GALT to acquire sufficient quantities of prions to establish infection. Without SI FDC-containing GALT, the gastrointestinal tract acts as a barrier against prion infection. While some prions may be delivered to the MLN (48, 49), the presence of the MLN in mice lacking SI GALT did not influence susceptibility, implying that the levels of prions delivered to MLN immediately after oral exposure are insufficient to establish replication. It has been suggested that prions are transcytosed into cecal patches by M cells (50), contrasting the absence of accumulation observed in this study. This discrepancy most likely relates to differing doses of prions (∼100 times greater than used here) and methods of administration used (gavage rather than feeding), potentially facilitating the uptake of a large bolus of prions into additional GALT compartments, which does not occur following exposure to a physiologically relevant dose via the oral cavity.

Following peripheral exposure, prions accumulate first in the draining lymphoid tissue (such as Peyer's patches in the SI after oral exposure) and subsequently spread to most other lymphoid tissues, including nondraining lymph nodes and spleen (3, 5, 26, 51). B cells recirculate between lymphoid tissues for several weeks (52) and often acquire FDC surface proteins during cognate antigen capture (53). We have shown that B cells recirculating between lymphoid tissues play an important role in the initial transfer of prions from the draining lymphoid tissue to other nondraining lymphoid tissues (51). The detection of PrP^Sc^ within LI GALT only at much later stages of disease is entirely consistent with secondary dissemination by B cells from the initial sites of infection in the GALT of the SI. The preferential migration of Peyer's patch-derived IgA-secreting plasmablasts to the SI, rather than to the LI (54), may enhance prion accumulation in the SI and restrict the early secondary dissemination to LI GALT.

After accumulating upon FDC, the prions are then amplified above the threshold required for neuroinvasion (3–5, 9, 55) and spread to the enteric nervous system and the CNS, ultimately causing neurodegeneration and death (9–11). Infection can spread to enteric nerves in SI GALT within 21 days of exposure (9), potentially in association with classical dendritic cells (56, 57). In the current study, no PrP^Sc^ was detected in the LI GALT by 15 weeks after oral exposure. Since the initial infection of enteric nerves occurs substantially before the detection of PrP^Sc^ in LI GALT (9), our data strongly support the conclusion that the LI GALT are not important early sites of prion neuroinvasion after oral exposure.

Many factors may exert an important influence on the host's susceptibility to oral prion infection. For example, the dramatically reduced susceptibility of aged mice to oral prion infection (58) coincides with a significant reduction in the number of mature M cells in Peyer's patches and disturbances to lymphoid tissue microarchitecture (59, 60). Conversely, chronic inflammation, through the formation of ectopic FDC-containing B-cell follicles (tertiary lymphoid tissues), may expand the tissue distribution of prions within infected hosts (61–63). It is plausible that damage to the LI mucosa and the associated immune pathology may also affect oral prion disease pathogenesis (64). Although intestinal helminth infections are common in animals and humans and cause significant morbidity in cattle, sheep, and goats, nothing was known about their effects on oral prion disease. Congruent infection with T. muris did not influence neuroinvasion or disease susceptibility irrespective of the time at which the mice were coexposed with prions, highlighting the important role of SI GALT in oral prion pathogenesis. Our data appear to contradict those in an independent study that reported that Salmonella enterica serovar Typhimurium-induced colitis exacerbated oral prion disease (65). However, while T. muris is restricted to the LI, subsequent data have shown that *S*. Typhimurium infection can also have a dramatic effect on M cells and classical dendritic cells in the SI (66, 67), which have key roles in oral prion pathogenesis (26, 27). This may have significantly influenced prion uptake in the SI, enhancing disease susceptibility independent of the effects on the LI.

In conclusion, our data demonstrate that the GALT in the SI, not the LI, are the major early sites of prion accumulation and neuroinvasion after oral exposure. This has important implications for our understanding of the factors that influence the risk of infection and the preclinical diagnosis of disease. Although LI GALT are not early sites of infection, the detection of PrP^Sc^ within the RAMALT and appendix has proved to be a useful method to detect prion-infected individuals during the preclinical phase (17, 18, 68) and has been used in the United Kingdom to gain insight into the possible prevalence of vCJD in the human population (19, 20). However, our data suggest that the time at which these tissues are sampled in relation to prion exposure may dramatically affect the sensitivity of these assays. For instance, humans with subclinical vCJD infection may have only minimal PrP deposition in appendiceal tissue (69). Together, these data show that analyses of such biopsy specimens may miss individuals in the early stages of oral prion infection and underestimate the disease prevalence.
